# Omi/HtrA2 Participates in Age-Related Autophagic Deficiency in Rat Liver

**DOI:** 10.14336/AD.2018.0221

**Published:** 2018-12-04

**Authors:** Jiahui Xu, Kun Jiao, Xin Liu, Qi Sun, Ke Wang, Haibo Xu, Shangyue Zhang, Ye Wu, Linguo Wu, Dan Liu, Wen Wang, Huirong Liu

**Affiliations:** Department of Physiology and Pathophysiology, School of Basic Medical Sciences, and Beijing Key Laboratory of Metabolic Disorders Related Cardiovascular Diseases, Capital Medical University, Beijing, China.; Department of Physiology and Pathophysiology, School of Basic Medical Sciences, and Beijing Key Laboratory of Metabolic Disorders Related Cardiovascular Diseases, Capital Medical University, Beijing, China.; Department of Physiology and Pathophysiology, School of Basic Medical Sciences, and Beijing Key Laboratory of Metabolic Disorders Related Cardiovascular Diseases, Capital Medical University, Beijing, China.; Department of Physiology and Pathophysiology, School of Basic Medical Sciences, and Beijing Key Laboratory of Metabolic Disorders Related Cardiovascular Diseases, Capital Medical University, Beijing, China.; Department of Physiology and Pathophysiology, School of Basic Medical Sciences, and Beijing Key Laboratory of Metabolic Disorders Related Cardiovascular Diseases, Capital Medical University, Beijing, China.; Department of Physiology and Pathophysiology, School of Basic Medical Sciences, and Beijing Key Laboratory of Metabolic Disorders Related Cardiovascular Diseases, Capital Medical University, Beijing, China.; Department of Physiology and Pathophysiology, School of Basic Medical Sciences, and Beijing Key Laboratory of Metabolic Disorders Related Cardiovascular Diseases, Capital Medical University, Beijing, China.; Department of Physiology and Pathophysiology, School of Basic Medical Sciences, and Beijing Key Laboratory of Metabolic Disorders Related Cardiovascular Diseases, Capital Medical University, Beijing, China.; Department of Physiology and Pathophysiology, School of Basic Medical Sciences, and Beijing Key Laboratory of Metabolic Disorders Related Cardiovascular Diseases, Capital Medical University, Beijing, China.; Department of Physiology and Pathophysiology, School of Basic Medical Sciences, and Beijing Key Laboratory of Metabolic Disorders Related Cardiovascular Diseases, Capital Medical University, Beijing, China.; Department of Physiology and Pathophysiology, School of Basic Medical Sciences, and Beijing Key Laboratory of Metabolic Disorders Related Cardiovascular Diseases, Capital Medical University, Beijing, China.; Department of Physiology and Pathophysiology, School of Basic Medical Sciences, and Beijing Key Laboratory of Metabolic Disorders Related Cardiovascular Diseases, Capital Medical University, Beijing, China.

**Keywords:** Omi/HtrA2, age, autophagy, liver

## Abstract

Liver is a vital organ with many important functions, and the maintenance of normal hepatic function is necessary for health. As an essential mechanism for maintaining cellular homeostasis, autophagy plays an important role in ensuring normal organ function. Studies have indicated that the degeneration of hepatic function is associated with autophagic deficiency in aging liver. However, the underlying mechanisms still remain unclear. The serine protease Omi/HtrA2 belongs to the HtrA family and promotes apoptosis through either the caspase-dependent or caspase-independent pathway. Mice lacking Omi/HtrA2 exhibited progeria symptoms (premature aging), which were similar to the characteristics of autophagic insufficiency. In this study, we demonstrated that both the protein level of Omi/HtrA2 in liver and hepatic function were reduced as rats aged, and there was a positive correlation between them. Furthermore, several autophagy-related proteins (LC3II/I, Beclin-1 and LAMP2) in rat liver were decreased significantly with the increasing of age. Finally, inhibition of Omi/HtrA2 resulted in reduced autophagy and hepatic dysfunction. In conclusion, these results suggest that Omi/HtrA2 participates in age-related autophagic deficiency in rat liver. This study may offer a novel insight into the mechanism involved in liver aging.

Omi/HtrA2, a mitochondrial serine proteaseprotein, is a member of the High Temperature Requirement (HtrA) family. Omi/HtrA2 which was originally identified as a heat-shock-induced serine protease in *Escherichia coli *[1-2], but now is known to be a mitochondrial pro-apoptotic protein and participate in mitochondrial homeostasis [3-4]. Previously, increasing evidences have linked Omi/HtrA2 to aging and the cellular protein quality control system. Loss of Omi activity was reported to be associated with aging-related diseases such as neurodegeneration and Parkinson’s disease (PD) in MND2 (motor neuron degeneration 2) mice [5]. In addition to neurodegeneration, loss of Omi/HtrA2 activity has been shown to cause premature aging [6-7]. The Omi/HtrA2 mutant mice showed an aging phenotype including small size, weight loss, heart enlargement, multiple tissue atrophy and early lethality [8]. The same premature aging phenotype was also found in the Omi/HtrA2 knockout mice, caused by deletion of the Omi/HtrA2 gene [9]. Aging is a physiological and inevitable process for all biological organisms. The incidence rates of chronic diseases, including metabolic syndromes, tumors and cardiovascular diseases, increase with aging [10]. All organs can be impaired by aging process, especially the liver [11]. Liver is a vital organ with many functions. Therefore, the maintenance of normal hepatic function is very important for health. The expression and distribution of Omi/HtrA2 varied among different organs, with high expression in the liver, brain, heart and kidney tissue [12]. These observations implied the possibility that Omi/HtrA2 may play a role in liver aging.

As one mechanism for removing old or dying cells, autophagy is also responsible for degrading intracellular misfolded proteins, redundant lipids and damaged organelles [13]. Autophagy can prevent harmful substances from accumulating and causing cellular damage [14]. Therefore, autophagy is essential for maintaining cellular homeostasis and plays an important role in ensuring normal organ function. Autophagic deficiency is considered to be among the principal reasons for aging [15]. Notably, in mammalian liver, autophagy is the main process for intracellular waste degradation [16]. The age-related decline in liver autophagy was observed [17], and enhancement of autophagy could protect cellular homeostasis and hepatic function in aging liver [18].

Many reports have shown that autophagy is involved in major fields of liver diseases [19]. Studies have discovered that decreases in the regulation sensitivity of autophagic proteolysis may be the cause for age-related decline in autophagy of hepatic cells, rather than decreases in autophagic proteolysis expression as originally hypothesized [20]. Therefore, the relationship between the decreased regulation sensitivity of autophagic proteolysis and aging liver needs to be further explored. A recent study has reported that the serine protease Omi/HtrA2 is a novel regulator of autophagy. Omi/HtrA2 can regulate both basal and stress-induced autophagy in neuronal cells [21], indicating that Omi/HtrA2 may be involved in autophagic deficiency of aging liver.

In this study, we would like to explore the relationship between the expression of Omi/HtrA2 and autophagy in rats’ liver, trying to understand the role of Omi/HtrA2 in the aging progress of liver. By doing so, we wish to offer a novel insight into the mechanism involved in liver aging.

## MATERIALS AND METHODS

### Animals

3-month-old (young group) SPF male Sprague Dawley (SD) rats and 9-month-old (adult group) SPF male SD rats were purchased from Beijing Vital River Experimental Animal Technology Co. Ltd, CHN. License number: SCXK (Beijing), 2012-0001. 22-month-old (aging group) SPF male SD rats were purchased from Changsha Tianqin Biological Technology Co. Ltd, CHN. License number: SCXK (Hunan), 2009-0012. Animal experiments were approved by the Animal Experiments and Animal Welfare Committee of Capital Medical University (Ethical Code: AEEI-2014-076).

### Ucf-101 treatment

Animals from the young and adult groups were randomized to receive vehicle or ucf-101 once at 48 hours before euthanasia (1.8 μmol/kg i.p, Merck, USA). Ucf-101 has been shown to inhibit 50-90% of Omi/HtrA2 activity at the tested concentrations [22-23]. All animals were fasted for 24 hours and then killed by euthanasia. The liver and blood were collected immediately.

### Measurement of serum parameters

Rats were anesthetized by intraperitoneal injection of 10% chloral hydrate aldehyde (0.03 ml/kg). Then blood was collected from abdominal aorta. Serum was separated by centrifugation (4°C, 3000 rpm, 10 min). All parameters were detected by 7180 automatic biochemistry analyzers (HITACHI, JPN).

### H&E staining

Liver tissue cubes (1 cm^3^) were fixed with 4% formalin and embedded in paraffin. 4 μm sections were stained with hematoxylin for 5 min and then stained with eosin for 5 min. After dehydration, the sections were observed and photographed using a Nikon microscope fitted with a camera (NIKON Corporation, JPN).

### Immunostaining

Liver tissue samples (1 cm^3^) were fixed with para-formaldehydeand embedded in paraffin. 4 μm sections were incubated with a dilution of rabbitanti-LC3 antibody (1:150, abcam, USA) and anti-Omi antibody (1:150, abcam, USA) antibody overnight at 4°C. Immuno-reactivity was detected using goat anti-rabbit IgG (zsgb, CHN). After dehydration, all sections were observed and photographed using a Nikon microscope fitted with a camera (NIKON Corporation, JPN).

### Immunoblotting

Liver tissues (100 mg) were lysed in 100 mL of RIPA lysis buffer in the presence of PMSF (10 mL). 25 μg of protein was separated on a SDS-PAGE gel and transferred onto a PVDF membrane. The membrane was blocked with 3% non-fat milk in PBS-T and incubated with rabbit anti-LC3 antibody (1:2000, abcam, USA), anti-Omi antibody (1:2000, abcam, USA), LAMP2 antibody (1:2000, abcam, USA) and Beclin-1 antibody (1:2000, abcam, USA) overnight at 4°C, respectively. The secondary antibody used was sheep anti-rabbit IgG-HRP antibody. The bands were detected using the ECL Plus Western blotting detection kit (Amersham, USA).

### EGFP-mRFP-LC3 assays

The mRFP (Red Fluorescent Protein)-GFP (Green Fluorescent Protein)-LC3 (Ad-tf-LC3, Hanbio, China) adenoviral particles were used to assess the maturation and amount of active autophagosomes [24-26]. Human normal liver cells QSG-7701 cultured on coverslips were transduced with Adenovirus tandem fluorescent mRFP-GFP-LC3 at 20 multiplicities of infection, 24 hours. Some cultured cells were first treated with 20 μM chloroquine (Sigma-Aldrich, C6628, St. Louis, MO, USA) for 30 min to block the autophagosome-lysosome fusion, then treated with 9.5μM ucf-101 for 48h with the continued presence of chloroquine. After above treatment, the cells were fixed with 4% paraformaldehyde, mounted and observed with a fluorescence microscope (ZEISS, Imager.A2, Baden-Württemberg, Germany). The number of GFP and mRFP dots was determined by manual counting of fluorescent puncta in random 5 fields of cells. Images from approximately 100 cells per treatment were collected and quantified for the number of red puncta (mRFPLC3 signal) per cell. The green fluorescent protein signal was recorded blindly, using the same exposure times and laser strength settings as for the red fluorescent protein channel. A yellow signal indicates not acidified, immature autophagosomes.

### Statistical analysis

Densitometry analyses of immunoblotting were performed using Image Lab 5.0 from BIO-RAD. Densitometry analyses of the immunostaining were performed using Image-pro plus 6.0 from Media Cybernetics. The data were analyzed using SPSS 17.0. Comparison between normal and ucf-101 groups was taken with independent sample T-test. Differences among different age groups were analyzed by one-way ANOVA followed by Student-Newman-Keuls method. Probabilities of 0.05 or less were considered statistically significant.

## RESULTS

### The morphology and function of liver declined during the natural aging process

**Figure 1.**
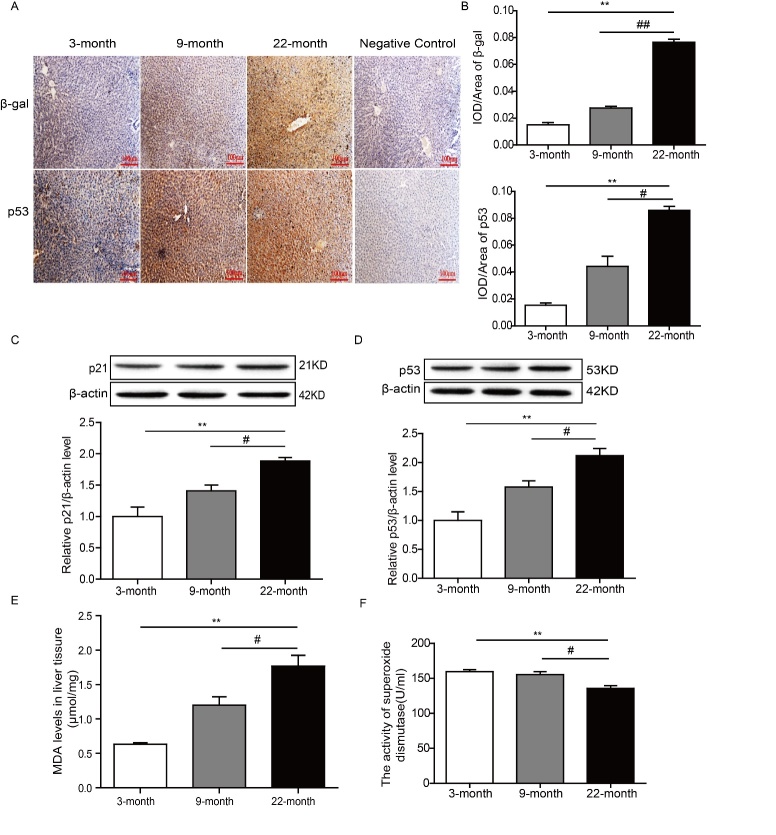
Both the level of aging-relative proteins and oxidative stress were increased in natural aging rat models. (A-D) The results of immunostaining and immunoblotting indicated that the expression of p53, p21 and β-gal was increased with growth of age. ^**^P<0.01*vs. *3 months, ^##^*P<*0.01 *vs.*9 month, ^#^*P<*0.05 *vs.*9-month, n=6-8. (E-F) Compared with 3 and 9-month groups, there were decreased SOD activity and increased MDA level in 22-month group. ^**^P<0.01*vs. *3 months, ^#^*P<*0.05 *vs.*9-month, n=6-8. SOD, Superoxide dismutase; MDA, Malondialdehyde.

Aging is a process with multifactor, complex and comprehensive physiological changes. We detected the expression of aging-related proteins, including p21, p53 and β-galactosidase to identify the senescence model. Both immune-histochemistry and immunoblotting results indicated that there was a gradually increasing tendency of aging-related proteins with the increasing of age (Fig. 1A-D). Oxidative damage to proteins was widely believed to be the primary cause of aging, so we also detected the level of oxidative stress. Compared with 3 and 9-month groups, there were decreased SOD activity and increased MDA level in 22-month group (Fig. 1E-F). Data above indicated the credibility of natural aging rats.

**Figure 2.**
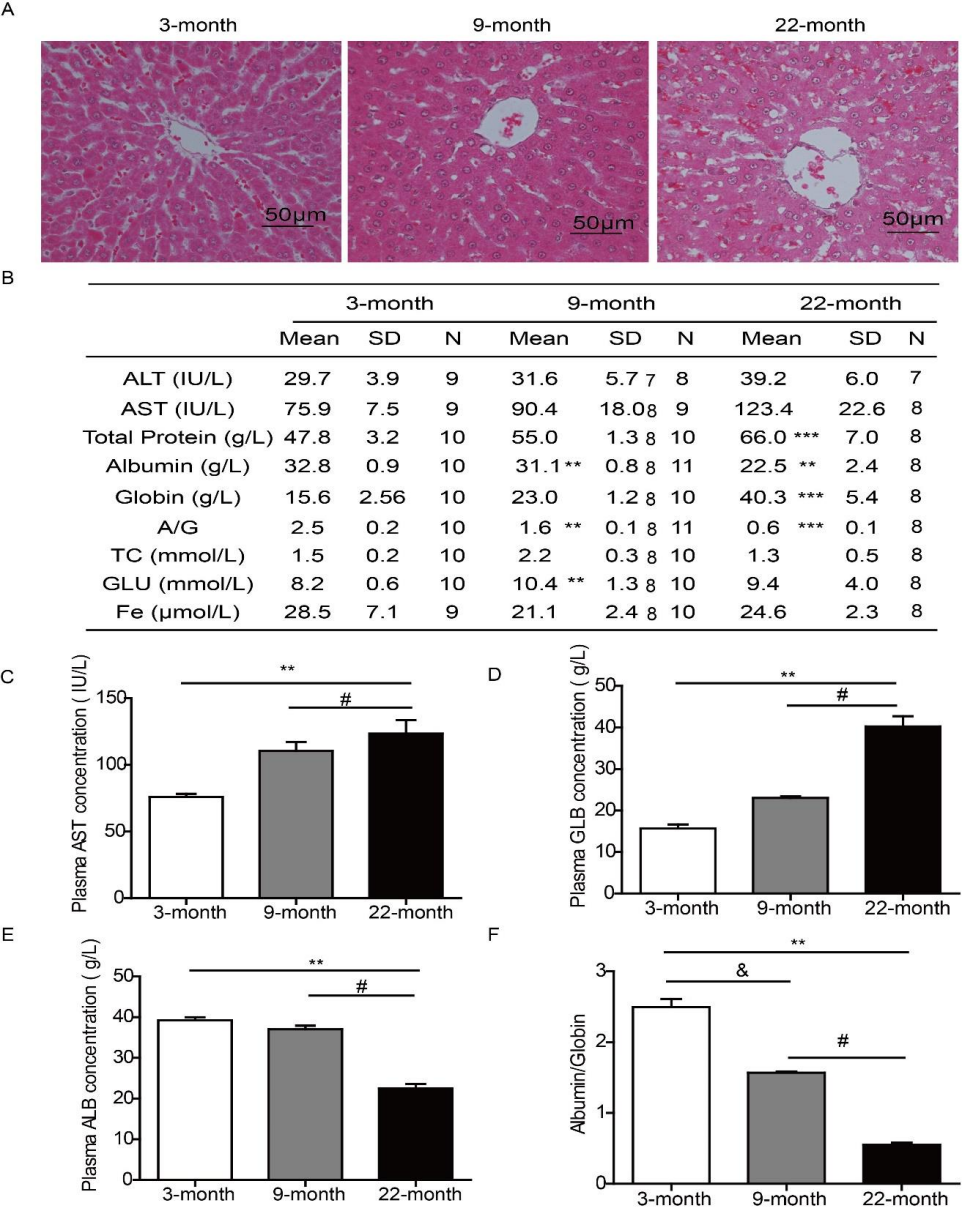
The morphology and function of liver were declined during the natural aging process. (A) The liver morphology of 3, 9, 22-month-old rats was detected by H&E staining, bar=50 μm. (B) The hepatic function was evaluated by serum biochemical detection. (C-F) With the growth of age, both AST and GLB were increased significantly, the level of ALB and the ALB/GLB ratio were decreased. ^**^P<0.01*vs. *3 months, ^#^*P<*0.05 *vs.*9 month, ^&^*P<*0.05 *vs.*3 month. n=8-10. ALB, albumin; GLB, globin; AST, aspartate aminotransferase.

To further understand the hepatic changes in aging process, we detected the morphology and function of liver. H&E staining was used to observe the liver tissue in young, adult and aging rats. The structure of hepatic lobules from young rats was clear, the hepatic cords were orderly, the cytoplasm was dyed pink and the nucleus was round with a prominent nucleolus. In contrast, in the liver from the aging rats, there was an unclear boundary in the hepatic lobule, with cytoplasmic staining loose, and binucleated cells were noted in greater numbers (Fig. 2A). Serum biochemical analysis is an important measure to evaluate the liver function (Fig. 2B). The results showed that, with increasing of age, both the aspartate aminotransferase (AST) and globin (GLB) levels were increased (Fig. 2C-D). Meanwhile the level of albumin (ALB) and the ALB/GLB ratio were decreased (Fig. 2E-F). Only produced by the liver, ALB is considered as the biomarker of hepatic function. Previously, a deceased ALB/GLB ratio (1.1), less than the normal level (1.3-2.5), has been noted in aging human populations [27]. Together, these above results indicated that hepatic function was declined during the aging process.

### Decreasing of Omi/HtrA2 was accompanied with hepatic dysfunction in the natural aging process

**Figure 3.**
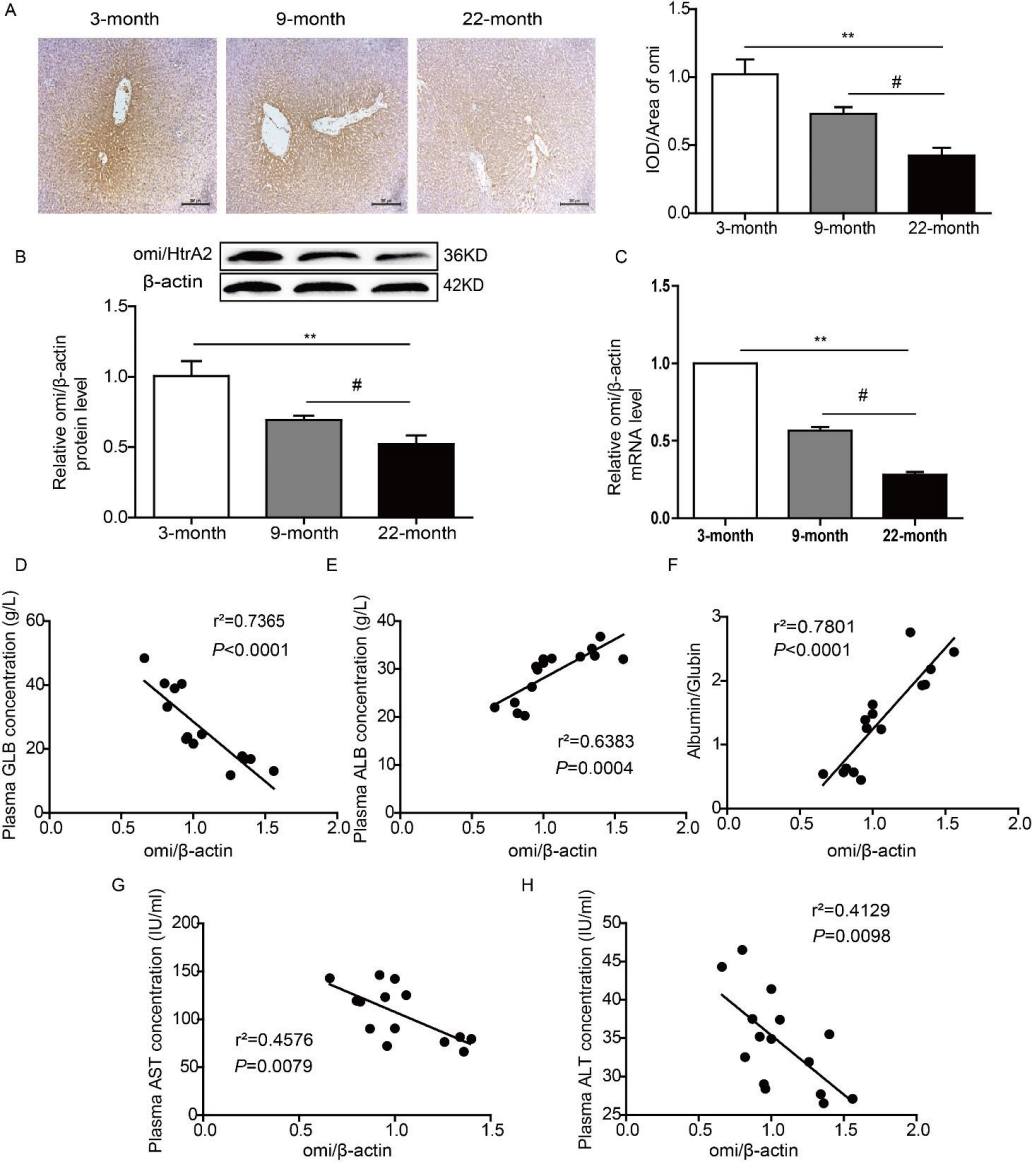
Decreasing of Omi/HtrA2 was accompanied with hepatic dysfunction in the natural aging process. (A) The protein level of Omi/HtrA2 in the liver of 3, 9, 22-month-old rats. Immunostaining, bar=200 μm. (B) Immunoblotting results indicated that the level of Omi/HtrA2 was decreased with growth of age. ^**^P<0.01*vs. *3 months, ^#^*P<*0.05 *vs.*9 month. n=6-8. (C) The mRNA level of Omi/HtrA2 was detected by quantitative RT-PCR. (D-H) Correlation analysis of Omi/HtrA2 and hepatic function. There was a positive correlation between the protein level of Omi/HtrA2 in liver and the index of hepatic function. ALB, albumin; GLB, globin; AST, aspartate aminotransferase; ALT, alanine aminotransferase.

The expression of Omi/HtrA2 in rats’ livers was measured by immunostaining, which demonstrated that Omi/HtrA2 was observed in all liver and localized mostly in hepatocytes near the portal area. Immunoblotting and RT-PCR for Omi/HtrA2 from liver extracts indicated that, in comparison to 3-month-old rats, the expression of Omi/HtrA2 was reduced in 9 and 22-month-old rats. In addition, both the mRNA and protein levels of Omi/HtrA2 decreased significantly in the 9 and 22-month-old rats compared to the 3-month-old rats. These results indicated that the level of Omi/HtrA2 in liver showed a progressive decline with increasing age (Fig. 3A-C). Additionally, a positive correlation was found between the level of Omi/HtrA2 in liver and index of hepatic function (Fig. 3D-H).

**Figure 4.**
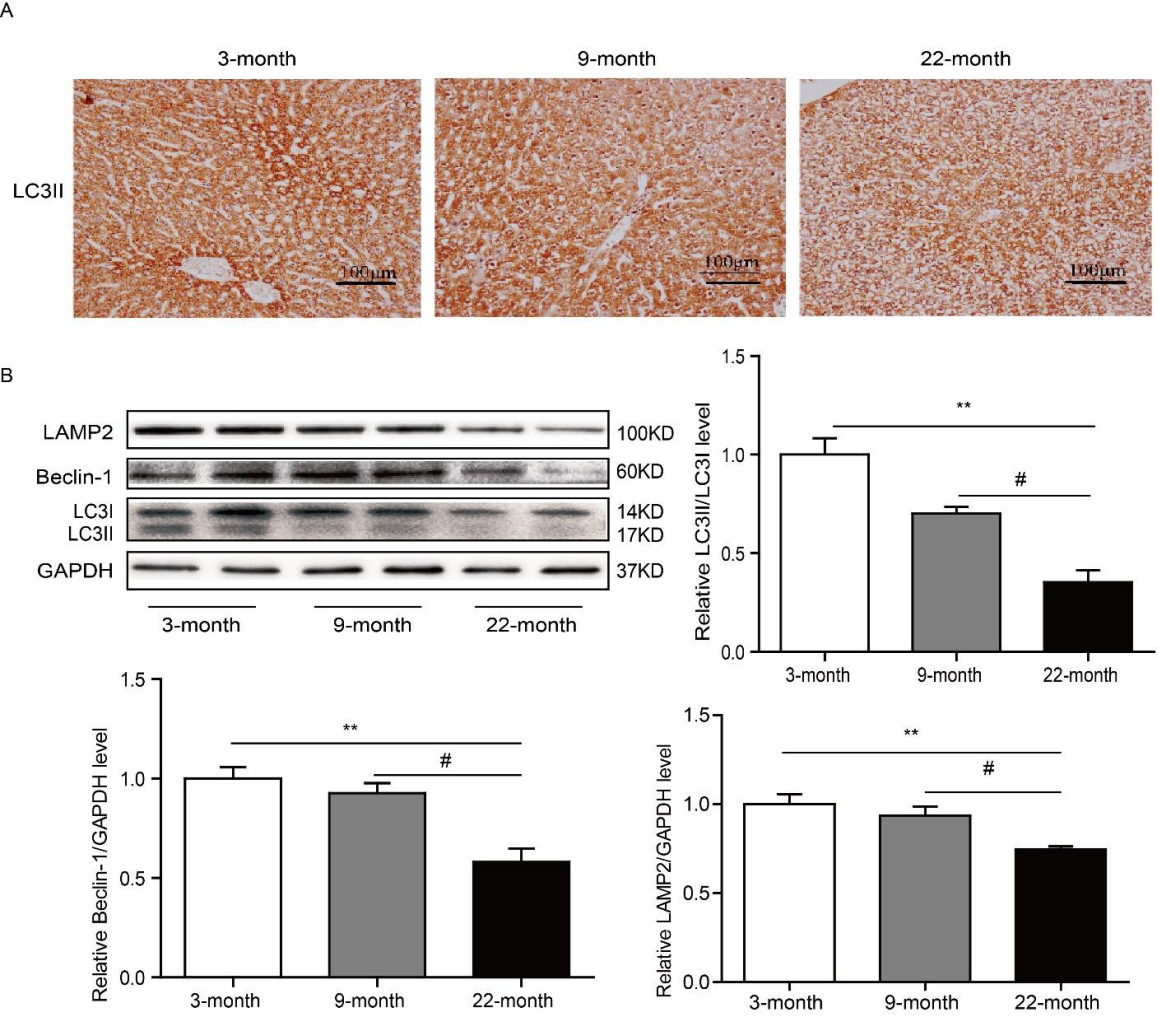
The level of autophagy in liver was decreased with aging. (A) The immunostaining of LC3II, bar=100 μm. (B) Immunoblotting results indicated that the level of autophagy was decreased with growth of age. ^**^P<0.01*vs. *3 months, ^#^*P<*0.05 *vs.*9 month. n=6-8.

### The level of autophagy in liver was decreased with aging

To further explore the relevance between Omi/HtrA2 and hepatic function in the normal aging process, we analyzed the autophagy level in aging liver. Microtubule-associated protein 1 light chain 3 (LC3) is involved in the formation process of the autophagosome. There are two molecular forms of LC3: LC3I and LC3II. LC3 exists as LC3I in the cell before autophagy occurs. As autophagy initiated, LC3I reacts with phosphatidy lethanolamine to form LC3II, which then migrates to the membrane of autophagosomes and is recognized as an autophagosomal marker [28]. The immunoblotting results showed that the level of LC3II was decreased significantly in liver of 22-month rats than 3 and 9-month rats (Fig. 4A). Containing a Bcl-2-homology (BH)-3 domain, Beclin-1 plays an important role in autophagy. When stimulated with stressor, the expression of Beclin-1 would increase and induce cell autophagy by interacting with the class III-type phosphoinositide 3-kinase (class III PI3K) [29-30]. The immunoblotting results indicated that the level of Beclin-1 was decreased significantly in liver of 22-month rats than 3 and 9-month rats (Fig. 4B). LAMP2 (Lysosome associated membrane protein 2) is a major protein component of the lysosomal membrane. Deficiency of LAMP2 can induce the accumulation of autophagic vacuoles [31]. The immunoblotting results showed that the level of LAMP2 was decreased significantly in 22-month rats than 3- and 9-months rats (Fig. 4B). All above data indicated that the level of autophagy in liver was decreased with aging.

**Figure 5.**
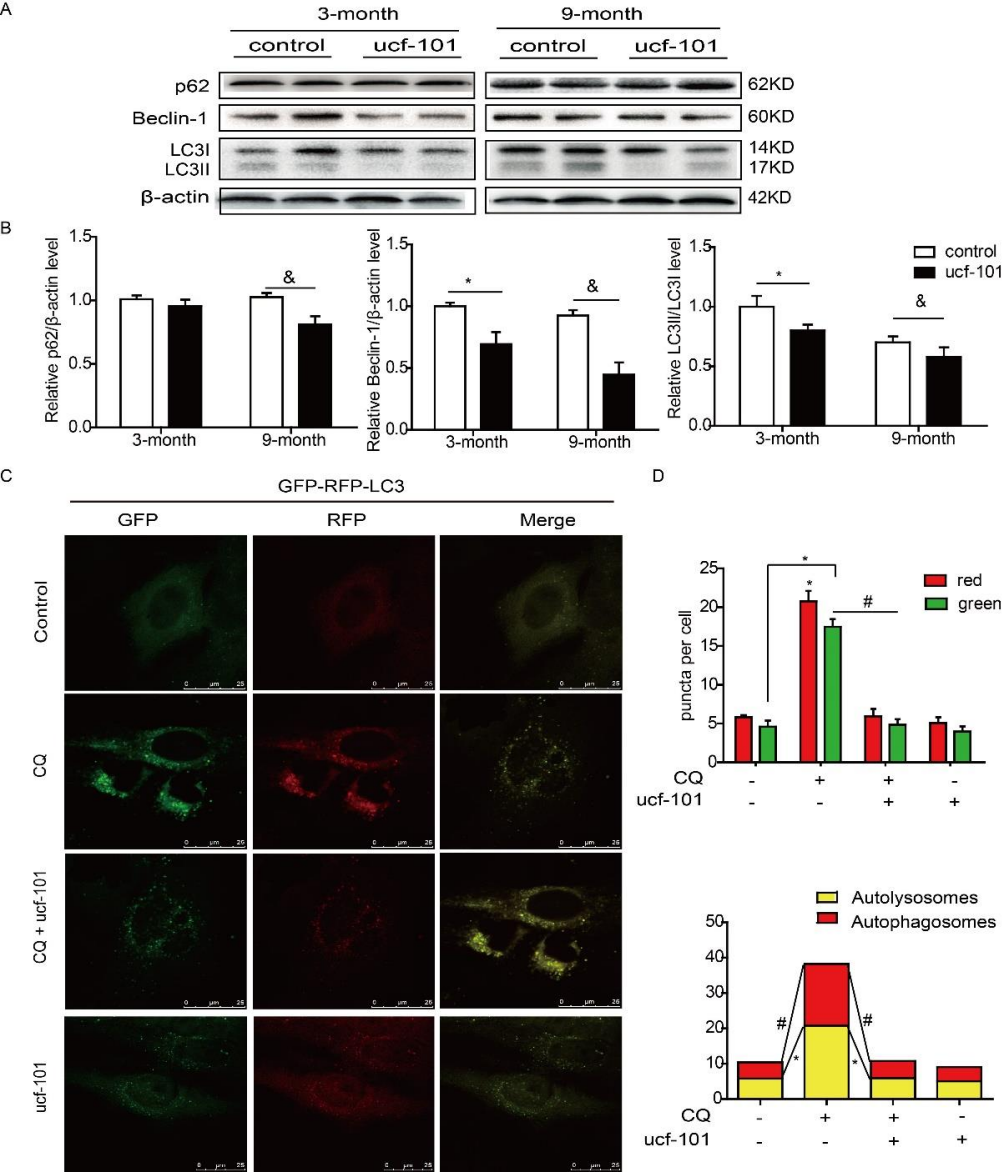
Inhibition of Omi/HtrA2 resulted in reduced autophagy in liver. (A-B) After treatment with ucf-101, the autophagy-related protein levels in 3 and 9-month-old rat’s liver were detected by immunoblotting. ^*^P<0.05 *vs. *3-month control, ^&^*P<*0.05 *vs. *9-month control. n=8-10. (C-D) The eGFP-mRFP-LC3 assays in vitro. The autophagy flux was reduced when cells were treated with ucf-101 (9.5μmol/L, 48h). Representative images showed LC3 staining in different groups of 7701 cells infected with GFP-RFP-LC3 adenovirus for 24 h. Acidified autophagosomes (red arrowheads in merged images) indicated active flux, yellow arrowheads pointed to immature autophagosomes. bar=25 μm. ^*^P<0.05, ^#^*P<*0.05. n=8. CQ, chloroquine.

**Figure 6.**
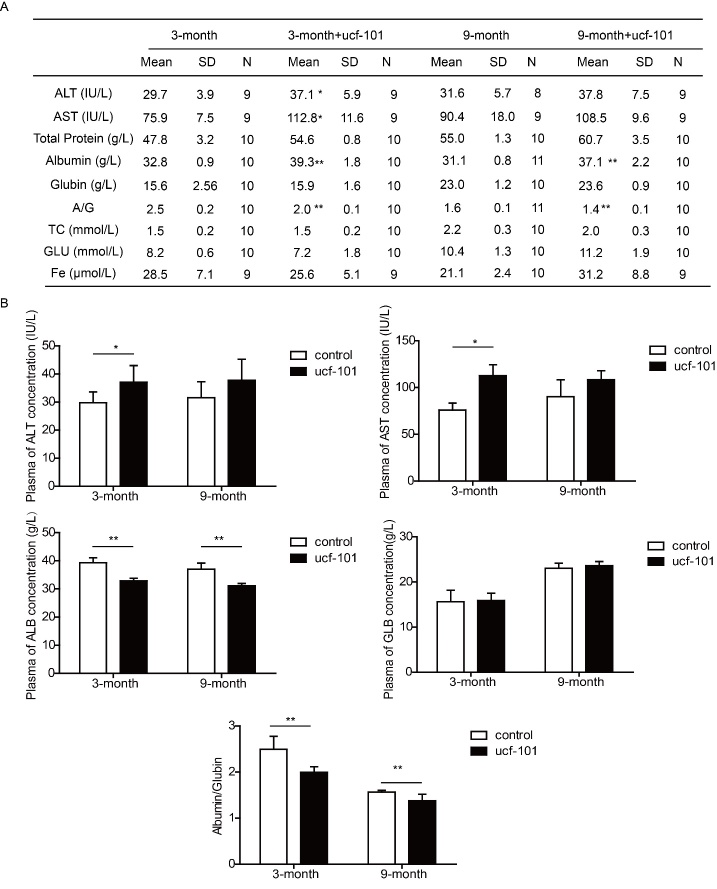
Inhibition of Omi/HtrA2 attenuated hepatic function. The effect of Omi/HtrA2 inhibitor ucf-101 on hepatic function from different rats was evaluated by serum biochemical detection. ^*^P<0.05, ^**^P<0.01. n=8-10.

### Inhibition of Omi/HtrA2 resulted in reduced autophagy in liver

In our study, the expression of LC3II was significantly increased in Omi/HtrA2-overexpressed H9C2 cells (Supplementary Fig. 1). To understand the effect of Omi/HtrA2 inactivation on hepatic autophagy, we tested the level of LC3II/I and Beclin-1 in rats’ liver after inhibition of Omi/HtrA2. The results showed that the level of LC3II/I and Beclin-1 were repressed after ucf-101 treatment in liver of rats from 3 and 9-month group, implying that the autophagy level was likely to be reduced by inhibiting Omi/HtrA2 activity (Fig. 5A-B). Furthermore, in cultured human normal liver cells QSG-7701, the tandem GFP-RFP-LC3 adenovirus was utilized to confirm the reduction of autophagy activity. The rationale of this assay is based on the pH sensitivity difference between the acidic autolysosome and the neutral autophagosome, which is exhibited by green fluroscent protein (GFP) and red fluroscent protein (RFP) to monitor the progression from autophagosome to autolysosome. When an autophagosome fuses with a lysosome to form autolysosomes, the GFP moiety degrades from the tandem protein, but RFP-LC3 maintains the puncta. The basic autophagy level was rather low after ucf-101 treatment, hence we first challenged the cells with chloroquine (CQ), by which to block the autophagosome-lysosome fusion, then we treated the cells with ucf-101 to evaluate the changes of autophagy activity. As shown in Figure 5C-D, after infection with the GFP-RFP-LC3 adenovirus, the cells showed both green and red fluroscent proteins. Ucf-101 treatment strongly reduced the number of red and green puncta of the tandem-fluorescent mRFP-GFP-LC3 autophagy biosensor, indicating an inhibition of autophagic flux in the human normal liver cells QSG-7701. All above data indicated that inhibition of Omi/HtrA2 resulted in reduced autophagy in liver.

### Inhibition of Omi/HtrA2 attenuated hepatic function

Finally, we primarily studied the relationship between Omi/HtrA2 and hepatic function. Our results indicated that ucf-101 treatment could lead to increased AST level, and decreased ALB/GLB ratio (Fig. 6A-B). To some extent, inhibition of Omi/HtrA2 had similar disadvantageous effects on hepatic function with aging.

## DISCUSSION

Omi/HtrA2, a serine protease, belongs to the HtrA family and can promote apoptosis through either the caspase-dependent or caspase-independent pathway. Omi/HtrA2 is a nuclear encoded protein with N-terminus mitochondrial targeting sequence and resides in the intermembrane space of the mitochondria as an immature form [3]. Omi/HtrA2 contains one or two C-terminal PDZ domains that activate its serine protease activity [32]. When exposed to apoptotic stimulus such as UV irradiation, Omi/HtrA2 is released into the cytoplasm from mitochondria and promotes apoptosis [33]. Previous studies have mainly focused on the function of Omi/HtrA2 in apoptosis [34]. Recently, Omi/HtrA2 was found to play an important role in maintaining cellular function [35]. However, the precise mechanism has not been elucidated yet. As reported, MND2 mice, in which the missense mutation in Omi/HtrA2 led to a remarkable loss of Omi/HtrA2 protease activity, appeared to be premature aging compared to normal mice [6]. Omi/HtrA2 KO mice show neurodegeneration and die after 4-6 weeks. Further investigations indicated that this phenomenon may be related with mitochondrial dysfunction and increased sensitivity to external stimuli through an unknown mechanism.

Liver is a vital organ with many functions, including metabolic regulation, detoxification, synthesis of protein and bile, etc [36]. Therefore, the maintenance of normal hepatic function is very important for health. Meanwhile, the morphology and function of the liver will deteriorate with aging. In this study, we examined the protein level of Omi/HtrA2 in liver from rats of different ages. The results indicated that there was a progressive decline in Omi/HtrA2 protein level with increasing age. Additionally, a positive correlation was found between Omi/HtrA2 protein level and hepatic function index. These results suggested decreasing of Omi/HtrA2 was accompanied with hepatic dysfunction in the natural aging process, similar to the phenotype observed in the MND2 mice [6].

Many studies have shown that the autophagy pathways are rendered inoperative in several age-related diseases [37]. Autophagy is essential for maintaining cellular homeostasis and play an important role in ensuring normal organ function. Autophagy can protect the liver from stress, and a basal level of autophagy is important for maintaining normal hepatic function [38]. Autophagic deficiency is considered to be among the principal reasons for aging [15]. Our study also suggested a correlation between the degeneration of hepatic function and a deficiency of hepatic autophagy. In this study, the level of several autophagy-related proteins, such as LC3II/I, Beclin-1 and LAMP2 in liver decreased significantly in aged rats than in young rats, indicating a decrease of autophagic activity with increasing age.

Currently three types of autophagy have been identified: macroautophagy, microautophagy and chaperone mediated autophagy. In physiological conditions, the major form of autophagy is macroautophagy, which functions to maintain cellular homeostasis [39]. As the most important organ related with metabolism, there are high concentration of reactive oxygen species in liver mitochondria. This renders hepatocyte organelles and other macromolecules easier to appear oxidative damage, which may activate autophagy pathways and then promote degradation of abnormal organelles and macromolecules. This mechanism helps to maintain cellular homeostasis and inhibits the decline of hepatic function [40]. Previously, a variety of studies have discovered that mice lacking Omi/HtrA2 exhibited symptoms of progeria and premature senescence, which was associated with deficiencies in autophagy [41]. Interestingly, in this study, the expression of Omi/HtrA2 in liver showed a similar trend with levels of autophagy-related proteins. In vitro, the progression from autophagosome to autolysosome was reduced by inhibiting the activity of Omi/HtrA2. Although our previous study had demonstrated that ucf-101 is highly selective to Omi/HtrA2 [2, 23], it was possible that ucf-101 might have additional effects on autophagy. To confirm the relationship between Omi/HtrA2 and autophagy, we transfected the QSG-7701 cells (the human normal liver cells) with small interfering RNA (siRNA) to konckdown Omi/HtrA2 gene expression. The results demonstrated that the autophagy level of liver cells was decreased with the reduction of Omi expression (Supplementary Fig. 2B). All above data indicated that inhibition of Omi/HtrA2 resulted in reduced autophagy in liver. Recent study has found that overexpression of Omi/HtrA2 in cells could also promote autophagy, possibly through interactions with HAX-1, which abolished the Beclin-1 antagonizing property [21]. This research suggests that Omi/HtrA2 may be involved in the regulation of autophagy in aging liver. Furthermore, the expression of autophagy-related proteins was reduced after inhibition of Omi/HtrA2 in young rats, with abnormal hepatic function. To some extent, inhibition of Omi/HtrA2 had similar disadvantageous effects on hepatic function with aging. Meanwhile, there is research also suggesting that the absence of Omi/HtrA2 or its inactivation can lead to mitochondrial dysfunction and increased mitophagy [42]. Therefore, the accurate mechanisms of Omi/HtrA2 involved in autophagy need to be further explored.

In conclusion, these results suggested that increased aging of rat livers was matched with a decrease in Omi/HtrA2 protein level, and there was a positive correlation between the level of Omi/HtrA2 and index of hepatic function. Furthermore, inhibition of Omi/HtrA2 resulted in abnormal autophagy and function in liver. These results suggest that the serine protease Omi/HtrA2 participates in age-related autophagic deficiency in rat liver. This study may offer a novel insight into the mechanism involved in liver aging.

## Supplementary data

Supplementary data is available online at www.aginganddisease.org/EN/10.14336/AD.2018.0221
