# Supplementary material for: Omi/HtrA2 Participates in Age-Related Autophagic Deficiency in Rat Liver
Source: Aging Dis. 2018 Dec 4;9(6):1031–42. doi: 10.14336/AD.2018.0221 (PMC6284766; doi:10.14336/AD.2018.0221)
Supplement: Supplementary file 1 — Supplementary data is available online at www.aginganddisease.org/EN/10.14336/AD.2018.0221 [file AD-9-6-1031-s.pdf]

SUPPLEMENTARY DATA

Omi/HtrA2 Participates in Age-Related Autophagic Deficiency in Rat Liver

Jiahui Xu#, Kun Jiao#, Xin Liu, Qi Sun, Ke Wang, Haibo Xu, Shangyue Zhang, Ye Wu, Linguo Wu, Dan Liu, Wen Wang*, Huirong Liu*

Department of Physiology and Pathophysiology, School of Basic Medical Sciences, and Beijing Key Laboratory of Metabolic Disorders Related Cardiovascular Diseases, Capital Medical University, Beijing, China.

© 2018. Xu J et al. Published online at http://www.aginganddisease.org/EN/10.14336/AD.2018.0221

SUPPLEMENTARY DATA

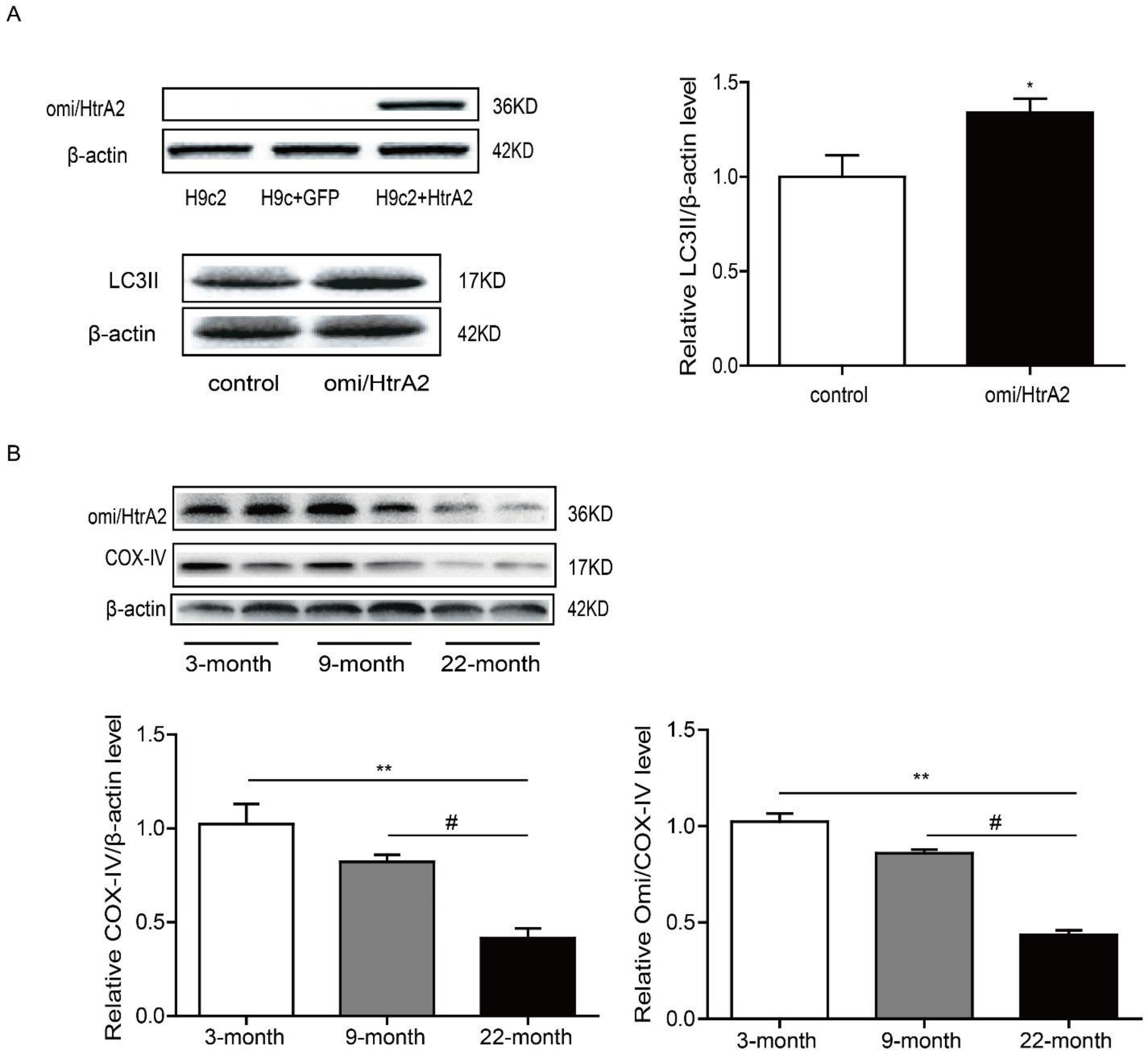

Supplementary Figure 1. (**A**) The expression of LC3II was significantly increased in Omi/HtrA2 overexpressed H9C2 cells. $^{*}P<0.05$. n=6. (**B**) The protein level of COX-IV was detected by immunoblotting, and the results indicated that the mitochondrial content decreased significantly in 22 months rats than 3- and 9-months rats. Furthermore, the relative Omi/COX-IV level was significantly decreased in 22 months rats. $^{**}P<0.01$, $^{\#}P<0.05$, n=8.

© 2018. Xu J et al. Published online at http://www.aginganddisease.org/EN/10.14336/AD.2018.0221

SUPPLEMENTARY DATA

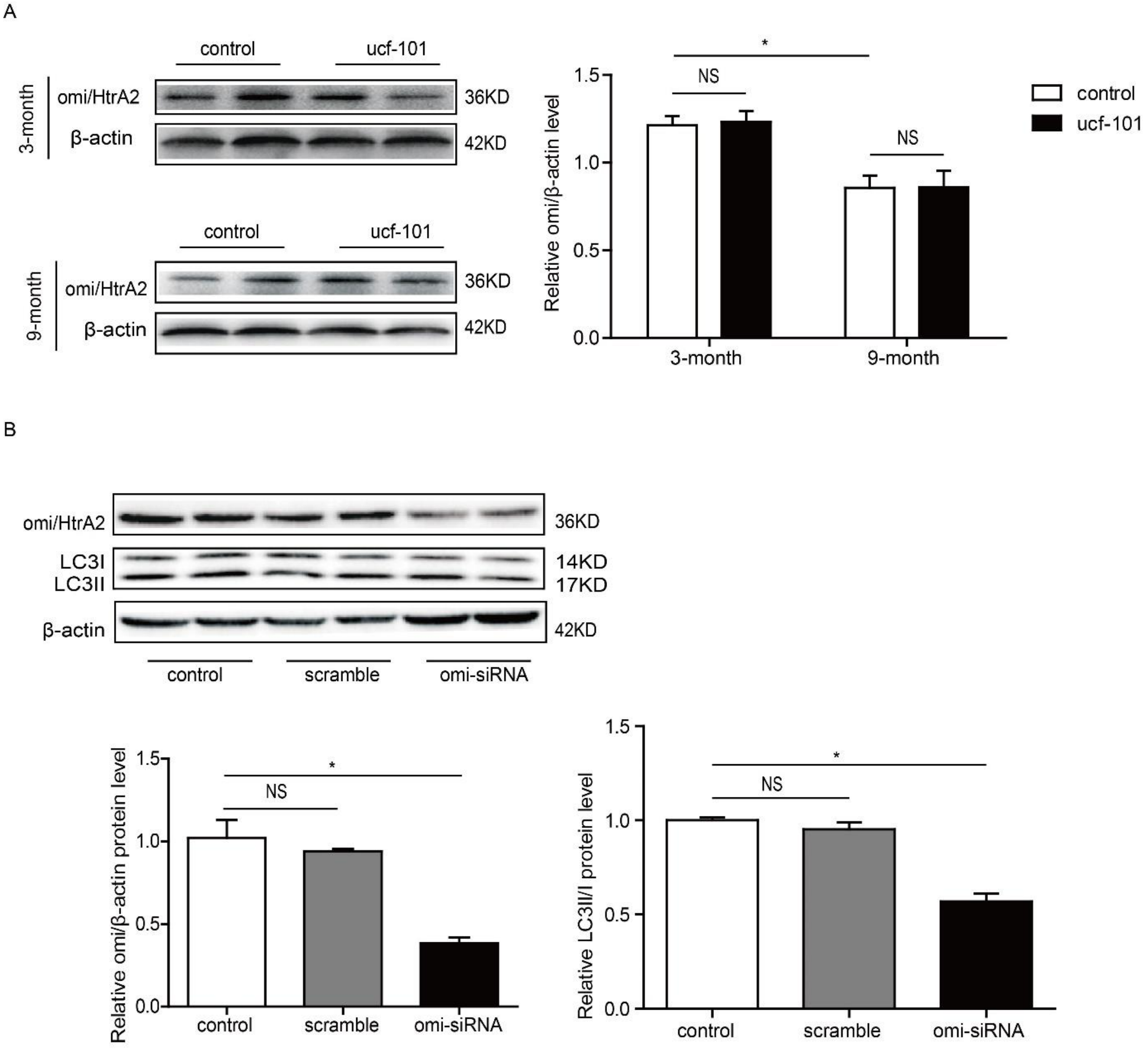

Supplementary Figure 2. (**A**) The effect of ucf-101 on Omi/HtrA2 protein level in rat liver. $^{*}P<0.05$, n=6. (**B**) The immunoblotting results indicated that both the protein level of Omi/HtrA2 and the level of autophagy were significantly decreased compared with the knockdown group. $^{*}P<0.05$, n=8.

© 2018. Xu J et al. Published online at http://www.aginganddisease.org/EN/10.14336/AD.2018.0221
